# Person-centred web-based support - development through a Swedish multi-case study

**DOI:** 10.1186/1472-6947-13-119

**Published:** 2013-10-19

**Authors:** Ulrika Josefsson, Marie Berg, Ingalill Koinberg, Anna-Lena Hellström, Margaretha Jenholt Nolbris, Agneta Ranerup, Carina Sparud Lundin, Ingela Skärsäter

**Affiliations:** 1 The Sahlgrenska Academy at University of Gothenburg, Institute of Health and Care Sciences, Box 457, SE-405 30 Gothenburg, Sweden; 2 Angered Hospital, Box 63, SE-424 22 Angered, Sweden; 3 School of Health Sciences, University of Borås, Borås, Sweden; 4 Department of Applied IT, University of Gothenburg, SE-412 96 Gothenburg, Sweden; 5 School of Social and Health Sciences, Halmstad University, Halmstad, Sweden

**Keywords:** Internet, Learning, Long-term illness, Person-centred care, Web-based support

## Abstract

**Background:**

Departing from the widespread use of the internet in modern society and the emerging use of web applications in healthcare this project captures persons’ needs and expectations in order to develop highly usable web recourses. The purpose of this paper is to outline a multi-case research project focused on the development and evaluation of person-centred web-based support for people with long-term illness. To support the underlying idea to move beyond the illness, we approach the development of web support from the perspective of the emergent area of person-centred care. The project aims to contribute to the ongoing development of web-based supports in health care and to the emerging field of person-centred care.

**Methods/Design:**

The research design uses a meta-analytical approach through its focus on synthesizing experiences from four Swedish regional and national cases of design and use of web-based support in long-term illness. The cases include children (bladder dysfunction and urogenital malformation), young adults (living close to persons with mental illness), and two different cases of adults (women with breast cancer and childbearing women with type 1 diabetes). All of the cases are ongoing, though in different stages of design, implementation, and analysis. This, we argue, will lead to a synthesis of results on a meta-level not yet described.

**Discussion:**

To allow valid comparisons between the four cases we explore and problematize them in relation to four main aspects: 1) The use of people’s experiences and needs; 2) The role of use of theories in the design of person-centred web-based supports; 3) The evaluation of the effects of health outcomes for the informants involved and 4) The development of a generic person-centred model for learning and social support for people with long-term illness and their significant others. Person-centred web-based support is a new area and few studies focus on how web-based interventions can contribute to the development of person-centred care. In summary, the main intention of the project outlined here is to contribute with both a synthesis of results on meta-level from four cases and a substantial contribution to the field person-centred care.

## Background

This paper outlines a multi-case research project that aims to develop and evaluate a person-centred model of web-based learning and support for people with long-term illness. The aim of the paper is to introduce the conceptual and procedural components of an overarching project and its potential contributions. The intentions presented here will draw the attention towards the ongoing development of internet use in the realm of private life as well as in health care.

Most people of all ages now have access to the Internet and to online alternatives for decision making. It is common to go online to seek not only information about medical or psychological treatments, but also help and support from people who are facing a similar situation (c.f) [1-4]. The Internet has also become an efficient alternative to traditional health care and treatment. There is scientific evidence for the benefits of some web-based therapies and computerized self-help treatments, including cognitive therapy for people with mood disorders [5], management strategies for patients with chronic disease (c.f) [6-8], and self-help for cancer patients [9]. However, many of these studies focus on the results for only one diagnosis, while the project outlined here examines four different cases and includes a variety of diagnoses, symptoms, and ways of providing web-based support. This, we argue, will lead to a synthesis of results on a meta-level not previously described.

Efficient web resources for people with illnesses must address their specific needs and experiences (c.f) [8,10,11]. To keep our focus on the person and on the idea of moving beyond the illness, we will approach the development of web support from the perspective of person-centred care (PCC). As a field, PCC is still under development [12], but studies have shown its positive outcomes for older people with long-term illness and their relatives [13,14]. Due to its emerging status there is no consensus on an absolute definition of PCC; however, a review of the concept [15] identified four major areas of research activity: (1) the extension of the scope of medicine from the purely biological to include psychological and social aspects; (2) the use of a 'patient-as-person’ view to understand the individual’s experience of illness; (3) the sharing of power and responsibility; and (4) the therapeutic alliance between the patient and the caregiver. PCC is thus characterized by its relational aspects, which make the patients’ experiences central. Patients’ interpretations of their illness and the surrounding circumstances will guide them in their recovery process, and thus the patients’ narratives are the starting point for PCC, built by a partnership between patients and their careers. This can lead to sharing of information, shared deliberation and shared decision-making in order to achieve commonly agreed goal [12,16].

Person-centred web-based support is a new area and few studies focus on how web-based interventions can contribute to the further development of PCC. This means there is a gap in the knowledge as to which person-centred learning and social processes mediated via the web can best strengthen patients’ abilities to make decisions about self-care and treatment options. In summary, the main intention of the project outlined here is to contribute with both a synthesis of results on meta-level from four cases and a substantial contribution to the field person-centred.

### Theoretical basis

A person-centred approach to the development of web-based support is an endeavour towards understanding the complex range of individual needs and knowledge processes. At the same time it is concerned with creating resources for change on individual, social, and societal levels. To capture and analyse this complexity we need to complement our person-centred approach with relevant conceptual tools and background theories that support a broad and flexible understanding. Three conceptual sources constitute our theoretical basis and inform our understandings of the phenomenon of web support on different levels and the relationships between those levels. At the same time these sources guide our understanding of core activities (such as seeking information and social support, learning, and participating in online communities) involved in the use and development of web-based support to manage long-term illness.

Our theoretical stance is based first, on the individual and social levels, on the ideas of *social theory of learning* as introduced by Wenger [17], who argues that social participation is a process of learning and knowing that includes the components of meaning, practice, community, and identity.

Second, also on the individual and social levels, and related to the ideas of learning, we apply the concept of *social support* as vital to everyday life an important contributor to mental and physical health and well-being [18]. House, Landis & Umberson [19] defined social support as the interactive process in which emotional concern, instrumental aid, information, and appraisal are obtained from one’s social network. The most common types of support in online communities seem to be informational and emotional support [20,21], which can offer stability and help members manage uncertainty while preserving their autonomy and integrity in social interactions [22].

Third, *the ecological model of systems theory*[23,24] serves as an overall theoretical structure that enables analysis and allows conclusions to be drawn from diverse interventional data in various contexts, and thereby increases understanding of the relationships within and between the different levels of change. The ecological model describes the interactions of systems in different settings, and it includes aspects of the environment that make it possible to take into consideration the multi-faceted nature of a person’s life course including such aspects as that person’s past and present, surroundings and social groups, and learning and development. The main levels of the system are the microsystem, mesosystem, exosystem, and macrosystem. These systems involve activities which interrelate both within themselves and with the other levels of systems [23,24].

## Methods/Design

### Ethics statement

The study protocol, covering data from four different studies, was approved by the ethics committee of the: Case 1: Regional Ethical Review Board in Gothenburg (Dnr/550-10 and 652–12); Case 2: Regional Ethical Review Board in Gothenburg (Dnr/368-07); Case 3: Regional Ethical Review Board in Gothenburg (Dnr/659-09) and Clinical Trials Protocol ID NCTO01565824; Case 4: Regional Ethical Review Board in Gothenburg (Dnr/762-08) and Clinical Trials Protocol ID A2007003. All participants gave written informed consent for recording, analysis and publication of their data collected within this study.

### Design

The research design uses a meta-analytical approach through its focus on synthesizing experiences from four Swedish regional and national cases of design and use of web-based support in long-term illness. The cases include children, young adults, and adults living with diagnoses such as bladder dysfunction and urogenital malformation, breast cancer, type 1 diabetes, and mental illness (Table 1). All of the cases are ongoing, though in different stages of design, implementation, and analysis.

**Table 1:** Characteristics of the four Swedish cases

	**Case 1**	**Case 2**	**Case 3**	**Case 4**
***Study group and setting***	Pre-school children (aged 4–6) with bladder dysfunction and urogenital malformation.	Women who have undergone surgery for breast cancer.	Women with type 1 diabetes who are pregnant or in early motherhood (with an infant up to 6 months old).	Young adults (aged 16–25) living with mental illness.
***Aim***	To investigate how a web-based model for person-centred learning and support affects health and self-esteem.	To investigate how an IT/web-based educational programme can support and affect health and well-being.	To investigate whether and how web-based support during pregnancy and early motherhood can improve well-being and diabetes management.	To develop and implement web-based support and treatment and investigate whether and how it can facilitate well-being.
***Intervention***	Web support with specially developed pictures and stories. Communication between children and a “web teacher” using Skype.	CD and web support with information and expert lectures on different topics (medical, social, and psychological).	Web support with information and a self-management tool for documentation and peer-support.	Web support for learning, self-care, and peer and professional support.
***Design***	Quasi-experimental trial.	Randomised, controlled trial.	Randomised, controlled trial.	Randomised, controlled trial.

The intention is to pursue a synthesis on a meta-level of these cases to create a model for the design and use of an effective person-centred web-based support for people with long-term illness. In some of these cases it may be possible to repeat the interventions, thereby adding a longitudinal dimension to the research design. Our approach contains several interventions and uses both qualitative and quantitative methods of data generation and analysis.

**Figure 1:**
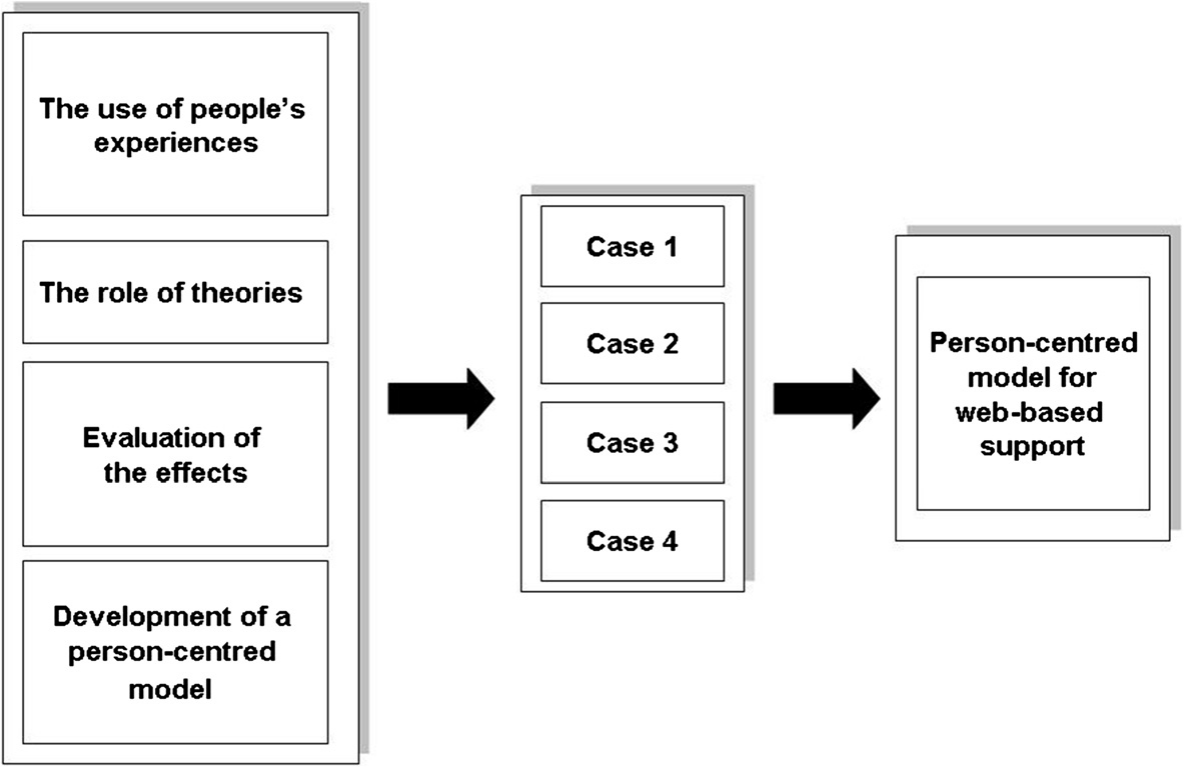
The studied aspects of web support development.

To allow valid comparisons between the four cases we intend to analyse and problematize them in relation to four main aspects (Figure 1): (1) *the use of people’s experiences*, (2) *the role of theories*, (3) *the evaluation of the effects*, and (4) *the development of a person-centred model*.

Our intention to contribute to the emerging field of PCC with web-based interventions based on people’s experiences motivates our focus on these four aspects; however, we acknowledge the need to analyse other aspects to make the picture of web-support development as complete as possible. Other aspects might include in-depth studies of design, different forms of technology, technology use, effects of web support for specific patient groups, and the larger role of web support in health care.

### The use of people’s experiences

Many patients have unique knowledge, based on their experiences of living with a disease for a long time. This knowledge is seldom used when decisions about patient care are taken, even though research indicates that shared decisions can help the patient to feel more in control and better satisfied, not only with the care, but with their whole attitude towards life [25]. The PCC approach to the development of web support is still in its infancy, and if patients’ participation is overlooked, there is a risk of losing important end-user information in the design [26,27], which could also jeopardize the efficient use of the support. A participatory approach to the development of web support is therefore important to both its development and eventual effectiveness. Efforts involve patients, their families, and their close friends to capture their experiences and needs when managing illness.

Therefore, in the four cases within the research project we explore and analyse peoples’ needs, wishes, and expectations to create person-centred web-based supports for learning and interaction in different contexts. Data collection includes various techniques including focus groups, films, web-based surveys, Skype and qualitative interviews with prospective users. Also, the design and evaluation of technology for such supports and self-monitoring must consider users’ different degrees and qualities of assimilating and using the technology in its actual context [27,28]. Hence, data is also gathered to identify the techniques used taken the variation of age, gender and diagnosis-related circumstances into account, and how and to what degree the web-based support is used. This means our work is characterized by an iterative design process, which includes phases of content specification followed by creative design tailored to the target audience, usability testing, and finally field testing.

### The role of theories

The design of computerized and other supports for patients is an important issue since it affects their capacities and roles [29]. The design might be affected by how different groups of actors participate in and influence the design process [30-32]. The design is also affected by more theoretical types of input. In a review of studies of various aspects of patient-decision supports, Durand et al. [33] conclude that not many of these (34%) consider the theories that informed the design or the evaluation. In a study of the design of web-based patient support, Elwyn et al. [34] suggest a model of how to organize the design process, including participating actors and specific steps. In contrast, Ekberg et al. [6] outline a model of the design of a web-based support explicitly aimed to be based on theories about learning and collaboration. However, neither of these studies applies a critical perspective to how theories are actually used in the design of web-based support or provide a summarizing perspective on theories and their different types and roles in the projects.

With this as a background, in our project we will investigate the use of theories in the design of PCC web-based support in two parts. In the first part we will categorize the different types of theories apparent in our four cases and their roles in the overall design of the projects. A preliminary step in this part is to distinguish between the role of broad background theories such as the social theory of learning, social support, and the ecological model of systems theory, theories that actually serve as a basis for the design of certain parts of the technology such as Communities of Practice [6], and theories affecting the organization of the design project such as Participatory Design [32]. An important result will also be the identification of actual instances of the theory types as they appear in the design of the web-based supports. The second part is to evaluate how the use of these types of theories in the four cases affect the web-based support in all of its aspects (e.g. design process, technology implementation, and health and other effects,) against the background of the PCC perspective. Our intention is to enhance the use of theories in the design of web-based support.

### Evaluation of the effects

In general, evaluation is recognised as crucial to the design of information technology (IT) solutions [35] as it creates space and opportunities for analysis and continuous improvements. There are many different models for evaluation and they cover different aspects of the particular IT (e.g. use, technology, economy, outcome, etc.) [36]. In the four cases within this project the evaluation model is characterized by evaluation of web support in real settings. This means a focus on real users facing real problems as opposed to evaluations in abstract settings in which researchers analyse theory, realistic scenarios, and assumed benefits [36].

Our evaluation concerns two main areas. First, it is concerned with personal benefits and health outcomes for the patients involved. This is an important aspect as PCC is based on a concern for individual needs and expectations. Since there are great variations in how people cope with illness it is important to understand who will benefit from using a web-based support and who might need other forms of support. Second, our evaluation focuses on how the benefits and health outcomes are actually created. This is important in order to form the basis to further develop and improve the web support in an iterativ design process. Additionally, the evaluation of the four cases will contribute to our efforts to introduce a person-centred model for the development of web-based support.

Data for evaluation will be generated through the online and offline instruments used in the cases. These measure different outcome measures in the four cases, such as health, well-being, quality of life, anxiety and depression, coping, self-efficacy, self-management, and knowledge about the long-term illness. However, to generalize the effect according to health according to the individual person, we assume that the use of the outcome measures well-being and self-efficacy, capturing from all four cases, will mirror the individuals’ subjective feeling of actual health situation as well as the persons’ view on how to cope with the stress situation according to health. Data will be analysed using both within-case and across-case statistical analyses.

### Development of a person-centred model for web support

Earlier work on the development of web support stresses the need for models that guide the design and adequately meet the needs of patients (c.f) [6,8,34]. However, most studies are based on single cases with a focus on only one disease or patient group. We acknowledge these as creditable contributions to a better understanding of the development of web supports, but they indicate a need for more generic approaches as well. Therefore, our multiple-case method, in which overlapping results from different contexts will provide comprehensive experiences, will contribute to the design of a more generally applicable, individually modifiable model.

Furthermore, the research design, with four cases, allows a synthesis of the experiences of both sexes with a variety of diseases, ages, phases of life, and contexts. These varied experiences will contribute to a model usable in everyday life that will facilitate person-centred decision making by patients with long-term illness and their significant others. Our assumption is that the participatory approach will promote a person-centred design, which will in turn contribute towards the effectiveness and usability of the synthesized web-based support model. We anticipate that this model will complement ordinary health care, and increase the quality, accessibility, participation, and feeling of control for patients and their relatives and friends. Our intention is that the fully developed model should be dynamic enough to be used in different conditions, contexts, and settings.

### Data collection

The multiple case method makes it possible to use different case experiences and environments, while also offering the opportunity to go beyond these [37]. Therefore, data collection includes various techniques such as focus groups, films, web-based surveys, and qualitative interviews with prospective users. Data is also gathered to identify the techniques used in the design of the web-based support, and how and to what degree they are used. In addition, data will include the theoretical concepts used in the different cases, how these concepts are used and applied, and the associated technical support available for the web-based technology in each case.

### Processing and analysis

Within-case analyses and across-case analyses will be performed on different levels [23,24]. The processing will also focus on social support [19], meaning-, practice-, community-, and identity-focused learning [17], and take into consideration variations of the studied phenomena [38]. Finally, data concerning level of person-centredness [12] will be assessed and analysed using statistical methods and qualitative methods at the meta-level.

This will be followed by evaluations of the usefulness of the web-based support using standard web-based instruments for measuring usability that have been redesigned to focus on person-centred issues. The outcome variables are the usability for different users to achieve their specified goals, and the effectiveness, efficiency, and user satisfaction in particular contexts of use [39]. The analyses will also include generative knowledge about variations between cases in relation to the design of a person-centred web-based support for long-term illness. Diffusion is partly built into the research approach through the multiple case study design, with a multiplicity of experiences rendering possible a meta-synthesis for the construction of a conceptual model of web-based support for patients with long-term illness. However, there will also be an analysis of the different measures of diffusion activities in the various cases.

## Discussion

In this paper we outline a research project with implications for persons suffering from long-term illness and for their significant others. There is a gap in the scientific knowledge of how to ensure that person-centred processes are transferred through web-based interactions. We argue that our multi-case approach will encourage a scientific renewal, bringing together different contexts of care, expanding our understanding, and potentially enabling patients and significant others to benefit from making more knowledge-based decisions.

The project we describe focuses on four main aspects of the development of person-centred web support: (1) *the use of people’s experiences*, (2) *the role of theories*, (3) *the evaluation of the effects*, and (4) *the development of a person-centred model*. Our aim to contribute to the emerging field of PCC motivates the focus on these aspects.

The conceptual sources that form our theoretical basis – social theory of learning, social support, and the ecological model of systems theory – offer various ways to understand the phenomenon of web-based support on different levels and the relationships between those levels. At the same time they guide our understanding of the core activities involved in the use of the Internet to manage long-term illness.

In our project continuing activities include an in-depth analysis of the use of people’s experiences in the development of web-based supports. In this next step we will problematize the use of people’s experiences when developing web-based support for long-term illness. We will pursue this in the light of participatory design principles, and the main questions will consider how people’s experiences are materialized in the development of person-centred web support and how their experiences *should* be materialized. However, although earlier work is characterized by the intention to use PD to capture patients’ needs, few studies analyse the development process and design results in relation to the emerging ideas of person-centred care. More specifically we will focus on issues such as the use of particular question areas and methods applied to capture people’s experiences. This involves an endeavour to understand what types of experiences are actually being captured in the process of developing a web-based support (e.g. personal experiences of patients, experiences from clinical work) and what this means in relation to PCC.

## Competing interests

The authors declare that they have no competing interests.

## Authors’ contributions

IS, MB, A-LH, AR, MJN, CS-L, IK were responsible for identifying the underlying structure of the research project. UJ drafted this manuscript and IS made the final version, which was reviewed by all authors. All authors read and approved the final manuscript.

## Authors’ information

For the University of Gothenburg Centre for Person-Centred Care (GPCC).

## Pre-publication history

The pre-publication history for this paper can be accessed here:

http://www.biomedcentral.com/1472-6947/13/119/prepub

## References

[B1] Bundorf MK Wagner TH Singer SJ Baker LC Who searches the internet for health information? Health Serv Res 2006 13 3 Pt 1 819 836 16704514 10.1111/j.1475-6773.2006.00510.x PMC1713205

[B2] Fox S Purcell K Chronic disease and the internet Pew Intern Am Life Proj http://www.pewinternet.org/Reports/2010/Chronic-Disease.aspx (2010 accessed August 1, 2013)

[B3] Kummervold PE Chronaki CE Lausen B Prokosch HU Rasmussen J Santana S Staniszewski A Wangberg SC eHealth trends in Europe 2005–2007: a population-based survey J Med Internet Res 2008 13 e42 19017584 10.2196/jmir.1023 PMC2629359

[B4] Pohjanoksa-Mäntylä M Bell JS Helakorpi S Närhi U Pelkonen A Airaksinen MS Is the internet replacing health professionals? a population survey on sources of medicines information among people with mental disorders Soc Psychiatry Psychiatr Epidemiol 2010 13 373 379 20225134 10.1007/s00127-010-0201-7

[B5] Vernmark K Lenndin J Bjärehed J Carlsson M Karlsson J Öberg J Internet administered guided self-help versus individualized e-mail therapy: a randomized trial of two versions of CBT for major depression Behav Res Ther 2010 13 368 376 20152960 10.1016/j.brat.2010.01.005

[B6] Ekberg J Ericson L Timpka T Eriksson H Nordfelt S Hanberger L Ludvigsson J Web 2.0 Systems supporting childhood chronic disease management. Designing guidelines based on information behaviour and social learning theories J Med Syst 2010 13 107 117 20433049 10.1007/s10916-008-9222-0

[B7] Timpka T Eriksson H Ludvigsson J Ekberg J Nordfeldt S Hanberger L eb 2.0 systems supporting childhood chronic disease management: a pattern language representation of a general architecture BMC Med Inform Decis Mak 2008 13 54 19040738 10.1186/1472-6947-8-54 PMC2627839

[B8] Ruland CM Holte HH Røislien J Heaven C Hamilton GA Kristiansen J Sandbaek H Kvaloy SO Hasund l Ellison MC Effects of a computer-supported interactive tailored patient assessment tool on patient care, symptom distress, and patients’ need for symptom management support: a randomized clinical trial J Am Med Inform Assoc 2010 13 403 410 20595307 10.1136/jamia.2010.005660 PMC2995659

[B9] Gustafson DH McTavish FM Stengle W Ballard D Hawkins R Shaw BR Jones E Julèsberg K McDowell H Chen WC Valrathongchai K Landucci G Use and impact of eHealth system by low-income women with breast cancer J Health Commun 2005 13 195 218 16377608 10.1080/10810730500263257

[B10] Nordfeldt S Hanberger L Malm F Ludvigsson J Development of a PC-based diabetes simulator in collaboration with teenagers with type 1 diabetes Diabetes Technol Ther 2007 13 17 25 17316094 10.1089/dia.2006.0053

[B11] Sparud-Lundin C Ranerup A Berg M Internet use, needs and expectations of web-based information and communication in childbearing women with type 1 diabetes BMC Med Inform Decis Mak 2011 13 49 21736713 10.1186/1472-6947-11-49 PMC3141376

[B12] Ekman I Swedberg K Taft C Lindseth A Norberg A Brink E Carlsson J Dahlin-Ivanoff S Johansson IL Kjellgren K Lidén E Öhlén J Olsson L-E Rosén H Rydmark M Stibrant Sunnerhagen K Person-centered care—Ready for prime time Eur J Cardiovasc Nurs 2011 13 248 251 21764386 10.1016/j.ejcnurse.2011.06.008

[B13] Edvardsson D Fetherstonhaugh D Nay R Promoting a continuation of self and normality: person-centred care as described by people with dementia, their family members and aged care staff J Clin Nurs 2010 13 2611 2618 20586833 10.1111/j.1365-2702.2009.03143.x

[B14] Gambling T Long AF The realisation of patient-centred care during a 3-year proactive telephone counselling self-care intervention for diabetes Patient Educ Couns 2009 13 219 226 20006458 10.1016/j.pec.2009.11.007

[B15] Mead N Bower P Patient-centredness: a conceptual framework and review of empirical literature Soc Sci Med 2000 13 1087 1110 11005395 10.1016/s0277-9536(00)00098-8

[B16] Ekman I Wolf A Olsson L-O Taft C Dudas K Schaufelberger M Swedberg K Effects of person-centred care in patients with chronic heart failure: the PCC-HF study Eur Heart J 2011 10.1093/eurheartj/ehr306 PMC3751966 21926072

[B17] Wenger E Communities of Practice: Learning, Meaning, and Identity 1998/2008 New York: Cambridge University Press

[B18] Albrecht TL Goldsmith DJ Thompson TL Thompson T, Miller K, Dorsey A, Parrott R **Social Support, Social Networks and Health** Handbook of Health Communication 2003 Hillsdale, NJ: Lawrence J. Erlbaum Associates 263 84

[B19] House JS Landis KR Umberson D Social relationships and health Science 1988 13 540 545 3399889 10.1126/science.3399889

[B20] Braithwaite DO Waldron WR Finn J Communication of social support in computer-mediated groups for people with disabilities Health Commun 1999 13 123 151 16370973 10.1207/s15327027hc1102_2

[B21] Preece J Ghozati K Rice RR, Katz JE Experiencing Empathy Online The Internet and Health Communication 2001 Thousand Oaks, CA: Sage 237 260

[B22] Rasmussen B Dunning P O’Connell B Young women with diabetes: using Internet communication to create stability during life transitions J Clin Nurs 2007 13 17 24 17518865 10.1111/j.1365-2702.2006.01657.x

[B23] Bronfenbrenner U Toward an experimental ecology of human development Am Psychol 1977 13 513 531

[B24] Bronfenbrenner U The Ecology of Human Development. Experiments by Nature and Design 1979 Cambridge, Mass: Harvard University Press

[B25] Mol A-M The Logic of Care. Health and the Problem of Patient Choice 2008 London and New York: Routledge

[B26] Eysenbach G Medicine 2.0: social networking, collaboration, participation, apomediation, and openness J Med Internet Res 2008 13 e22 18725354 10.2196/jmir.1030 PMC2626430

[B27] Kreps GL Neuhauser L New directions in eHealth communication: opportunities and challenges Patient Educ Couns 2010 13 329 336 20202779 10.1016/j.pec.2010.01.013

[B28] Storni C Multiple forms of appropriation in self-monitoring technology: reflections on the role of evaluation in future self care Int J Hum Comput Interact 2010 13 5 q537 q561

[B29] Josefsson U Ranerup A Consumerism revisited: the emergent roles of new electronic intermediaries between citizens and the public sector Inf Polity 2003 13 167 180

[B30] Høstgaard AM Bertelsen P Nøhr C Methods to identify, study and understand end-user participation in HIT development BMC Med Inform Decis Mak 2011 13 57 21955493 10.1186/1472-6947-11-57 PMC3196903

[B31] Lehoux P Hivon M William-Jones B Urbach D The worlds and modalities of engagement of design participants: a qualitative case study of three medical innovations Des Stud 2011 doi:10:1016/j.ddestud.20111.01.001

[B32] Spinuzzi C The methodology of participatory design Tech Commun 2005 13 163 174

[B33] Durand A-M Stiel M Boivin J Elwyn G Where is the theory? Evaluating the theoretical frameworks described in decsion support technologies Patient Educ Couns 2008 13 125 135 18242040 10.1016/j.pec.2007.12.004

[B34] Elwyn G Kreuwel I Durand A-M Sivell S Joseph-Williams N Evans R Edwards A How to develop web-based decision support interventions for patients: a process map Patient Educ Couns 2011 13 260 265 20627644 10.1016/j.pec.2010.04.034

[B35] Hevner A March S Park J Ram S Design science in information systems research MIS Q 2004 13 75 105

[B36] Pries-Heje J Baskerville R Venable JR Strategies for design science research evaluation 2004 87 Galway, Ireland: Proceedings of the 16th European Conference on Information Systems 255 266

[B37] Yin RK Case Study Research. Design and Methods 2003 3 Thousand Oaks, CA: Sage Publications

[B38] Marton F Ming Fai P Two Faces of Variation Proceedings of the 8th European Conference for Learning and Instruction 1999 Gothenburg, Sweden: Gothenburg University

[B39] Gulliksen J Göransson B User-centred systems. [In Swe: Användarcentrerad systemdesign.] 2002 Studentlitteratur: Lund

